# How Insightful Is ‘Insight’? New Caledonian Crows Do Not Attend to Object Weight during Spontaneous Stone Dropping

**DOI:** 10.1371/journal.pone.0167419

**Published:** 2016-12-14

**Authors:** P. D. Neilands, S. A. Jelbert, A. J. Breen, M. Schiestl, A. H. Taylor

**Affiliations:** 1 School of Psychology, University of Auckland, Auckland, New Zealand; 2 Department of Psychology, University of Cambridge, Cambridge, United Kingdom; 3 School of Biology, University of St Andrews, St Andrews, United Kingdom; 4 Max Planck Institute for the Science of Human History, Jenna, Germany; Consiglio Nazionale delle Ricerche, ITALY

## Abstract

It is highly difficult to pinpoint what is going through an animal’s mind when it appears to solve a problem by ‘insight’. Here, we searched for an information processing error during the emergence of seemingly insightful stone dropping in New Caledonian crows. We presented these birds with the platform apparatus, where a heavy object needs to be dropped down a tube and onto a platform in order to trigger the release of food. Our results show New Caledonian crows exhibit a weight inattention error: they do not attend to the weight of an object when innovating stone dropping. This suggests that these crows do not use an understanding of force when solving the platform task in a seemingly insightful manner. Our findings showcase the power of the signature-testing approach, where experiments search for information processing biases, errors and limits, in order to make strong inferences about the functioning of animal minds.

## Introduction

When faced with a difficult problem, humans can often spend a prolonged period of time trying to solve the problem without success, only for the solution to arrive suddenly and unexpectedly, often accompanied by a subjective ‘aha’ moment. Such ‘insightful’ problem solving is an implicit process. where the problem is restructured following the impasse [1,2], though it is not yet clear exactly what cognitive mechanisms humans use during this process [3,4]. Since Kohler’s research with apes at the beginning of the 20^th^ century [5], there has been debate over whether or not animals are also capable of insight [1,6,7]. How insight is defined in the animal cognition literature varies; one widely used definition, from Thorpe [8], states that insight occurs with “the sudden production of a new adaptive response not arrived at by trial-and-error behaviour”, while more recent definitions are more mechanistic, emphasising the importance of concepts such as mental models, means-end understanding, and causal knowledge in producing insightful behaviour [9–11].

A number of different bird behaviours fit these definitions of insight, including string pulling [11], solutions of the Aesop’s fable task [6] and solutions of the von Bayern paradigm [12]. In the string pulling task, birds have to pull up a string which is hanging from a perch in order to obtain the reward at the end of it. Some birds, such as keas [13], neo-tropical parrots [14], and corvids [11], can solve this task on their first trial. This fits Thorpe’s definition, as adaptive behaviour is produced without any evidence of trial and error learning. However, an alternative explanation for this success is a perceptual-motor feedback loop [15]. Pulling and stepping on the string can act as a reinforcer, as it moves the food closer to the bird, and so provides reinforcement for the bird to repeat these behaviours. In one experiment exploring this alternative explanation [15], the crows’ visual access to the food was restricted, and in another, the strings were laid horizontally and looped so that the initial pulls on the strings did not move the food [16]. In both experiments, the feedback loop was disrupted and the crows’ performance on the task was drastically reduced.

Another example of potential animal insight is the Aesop’s Fable paradigm, where animals have to raise the water level in a container via displacement in order to obtain a reward. Chimpanzees and orang-utans have been shown to solve this task by spitting additional water into the container [17,18], whilst rooks dropped stones into the container in order to raise the water level [6]. The cognitive mechanisms behind both the apes’ and rooks’ success are unclear. For the apes, it is difficult to know whether they planned a solution based on a causal understanding of displacement or tried out various behaviours in their behavioural repertoire until they happened upon a solution. For the rooks, it is unclear if subjects were not simply repeating a learned response, given they had learnt to stone-drop in a previous experiment [19].

Solution of the von Bayern paradigm is also an example of seemingly ‘insightful’ behaviour. Similarly to the results of the Aesop’s fable experiments, after receiving experience of pushing down a platform with their beak, New Caledonian crows will then drop stones down a tube positioned above the platform in order to trigger it [12]. This spontaneous innovation of stone dropping can be described as ‘insightful’ as it emerges without trial and error learning and so fits Thorpe’s definition. Two hypotheses have been proposed to explain the crows’ performances [12,20,21]. One possibility is that the birds use an understanding of force; that is, they learn from pushing the platform with their beak that pressure needs to be applied to the platform for it to trigger. By coupling this understanding with knowledge that heavy, falling objects also exert sufficient force, the birds realise that stone dropping gains them the out-of-reach food. An alternate possibility is that the birds learn that contact between their beak and the platform leads to it triggering. They then attempt to recreate this contact by dropping an object external to their body onto the platform.

As can be seen from the above studies, there are a number of competing hypotheses for many recent examples of animal ‘insight’. This has led to a growing call for comparative psychologists to move beyond describing seemingly ‘insightful’ behaviour in non-human animals [19,22,7,23]. Instead it has been suggested that researchers attempt to pinpoint the actual cognitive mechanisms being used by an animal during problem solving [21,24–26]. One powerful way to do this is to use the ‘signature–testing approach’ [26]. Inspired by Alan Turing’s work on machine intelligence [27], this attempts to make inferences about thought processes by not only examining problem solving successes, but also the information processing biases, errors and limits made by an individual. If a potential cognitive mechanism does not predict the presence of these observed signatures it is unlikely to be generating the behaviour in question. Thus the presence and absence of these signatures constrains the type of cognitive mechanism that can be producing behaviour, allowing for stronger inferences to be made about the type of cognitive process used by an animal during problem solving.

One particular cognitive signature, the ‘weight inattention error’, can be used to test between the two hypotheses explaining crows’ ‘insightful’ performances on the von Bayern paradigm. The contact hypothesis predicts that crows should be insensitive to object weight as their understanding of the task is based only on the need for contact between an object and the platform. In contrast, if the crows have an understanding of force, they should understand that the weight of an object is important for successfully collapsing the platform and so prefer heavy objects to light objects when innovating stone dropping. Therefore, determining whether this information processing error is present or absent in New Caledonian crows is a powerful way to explore the cognitive mechanisms behind crows’ ‘insightful’ solutions of this task. In this study, we first gave New Caledonian crows experience of pushing a platform with their beaks, as in von Bayern et al [12], before examining whether our subjects chose heavy, functional objects when they began spontaneously dropping stones onto a platform, or if they made the weight inattention error.

## Methods

### Ethics statement

This study was conducted under approval from the University of Auckland ethics committee (reference no. R602). The Province Sud granted us permission to work on Grande Terre, New Caledonia and to capture and release crows (Permit No.2962-2015/ARR/DENV). All birds were released at their site of capture at the end of testing.

### Subjects

Subjects were twelve wild-caught New Caledonian crows, captured from various sites across Grande Terre. The birds were housed in an eleven-cage outdoor aviary before being released. All cages were at least 2m^2^ x 3m. The crows were housed in the aviary on three separate occasions. The first group were housed in the aviary from April to November 2014, the second group were housed in the aviary from April to August 2014 and the final group were housed there from April to August 2016. All crows were captured by whoosh nets. The area around the whoosh net was baited until groups of crows were feeding regularly on it. The whoosh net was then released when a family group was present. Sexing and ageing were carried out using methods from Kenward et al, 2004 [28] Six of the twelve crows were adults. Nine of the birds were male: four juveniles and five adults. Three of the birds were female: two juveniles and 1 adult (see Table 1 for details.) Crows were fed a diet of papaya, meat, dog biscuits and egg daily, and had access to water *ad libitum*.

**Table 1:** Crows’ pre-test block preferences.

Identity	Adult or Juvenile	Sex	Number of trials	Number of heavy block choices
D4B	Adult	Male	20	6
D4R	Juvenile	Female	20	10
D3R	Juvenile	Female	20	10
D3B	Juvenile	Male	20	9
RWY	Adult	Male	20	8
Sort	Adult	Male	20	9
Svart	Juvenile	Male	20	8
Den	Juvenile	Male	20	10
Black	Juvenile	Male	20	9
Nero	Adult	Male	20	9
Preto	Adult	Male	20	9
Noir	Adult	Female	20	15*

The number of times across 20 trials that the crows picked the tube containing heavy blocks to obtain the reward.

*p<0.05

### Materials

**Fig 1:**
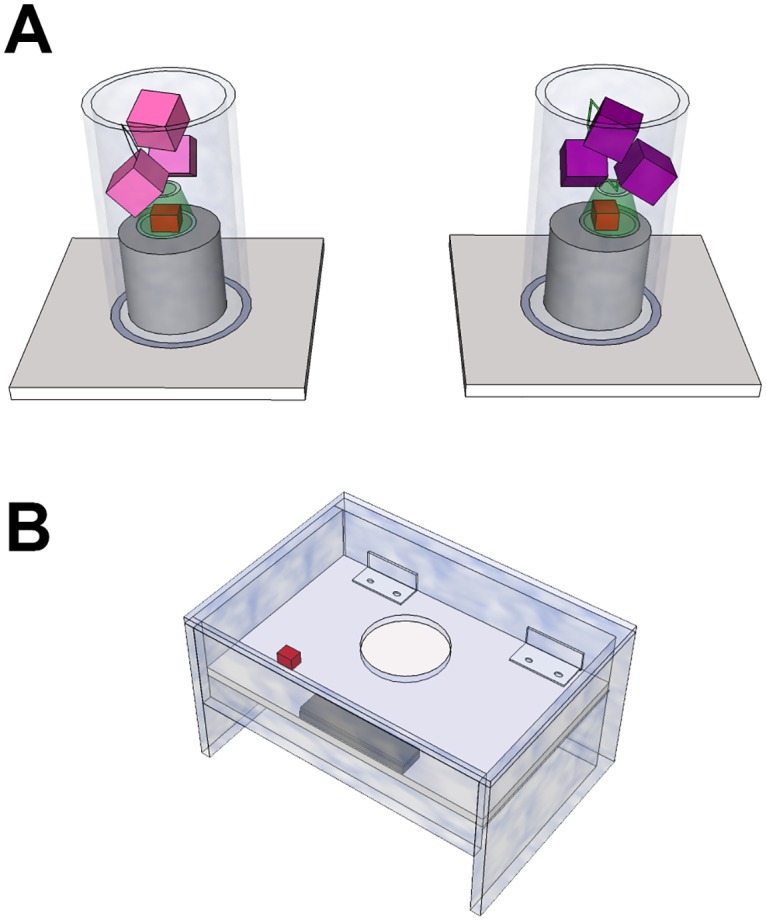
Diagram of platform apparatus and object pre-handling pretest apparatus. A. Illustrates the set up for object pre-handing pretest. One tube contained heavy blocks and the other contained light blocks. The birds had to pull the blocks out of the tube in order to retrieve the reward. Birds were allowed to obtain the reward from one tube. If birds had a preference for the heavy or light blocks, they should approach the corresponding tube at above chance levels. B. The platform apparatus. When the birds pecked the platform, it collapsed and the birds could access the food.

The platform apparatus was a Perspex box (180x110x85mm) with a 90mm tube (inner diameter = 40mm) on top and a platform inside held up by a magnet. This apparatus functions as follows: when a heavy object is dropped down the tube it falls onto the platform, which causes the platform to collapse and the meat sitting on the platform to fall out of the apparatus (Fig 1B). The blocks were 30mm x 20mm x 10mm, weighed either 15g or 1g and were coloured either purple or pink. These colours were chosen because crows are tetrachromatic and so can discriminate colour [29–31], the colours pink and purple did not have any obvious ecological correlates which may induce preference or aversion, and because past work had shown the crows could distinguish between them (AHT pers. obv.) The plastic tubes used to habituate the crows to moving the blocks were 120mm x 120mm x 60mm, while the Perspex tubes used for the object preference pre-test were 150mm high, with an inner diameter of 40mm.

### Procedure

Birds were randomly allocated to two groups. For Group 1 the purple blocks were heavy across all stages of our experiment, while for Group 2 the pink blocks were heavy. While the heavy blocks were of sufficient weight to trigger the platform apparatus, the light ones were not.

### Habituation

Crows were habituated to the purple and pink blocks by placing food underneath two blocks, one of each colour. Habituation continued until neophobic responses towards the objects stopped. They were then habituated to moving the blocks with their beaks by placing the blocks in clear plastic containers with food underneath so that the birds had to move the blocks out of the way to access the food.

### Object handling pre-test

To ensure the crows had no preference to handle blocks of one colour or weight we gave them 20 object handling trials. Here, food was placed in a plastic bottle top with a 3cm handle. This food holder was then placed in a vertical, Perspex tube and three blocks of one colour were stacked on top of it (Fig 1A). Crows had to pull three blocks out of the tube to be able to pull the container out by its handle and so gain the food. A second tube was set up 30cm away, with three blocks of the other colour stacked on top of the baited container. Block type was pseudo-randomised between tubes, across trials. While both tubes were always baited, the crows were only allowed to gain food from one tube. If the crows interacted with one tube and then attempted to interact with the other tube, the experimenter entered and removed the second tube before the crow could access the food. If crows had a preference for objects of a particular colour or weight, we expected them to choose the tube containing the objects they preferred at above chance levels.

### von Bayern platform pushing procedure

Crows were tested following the methodology outlined in von Bayern *et al*. [12]. The birds were habituated to the platform apparatus by placing meat beside and on top of the apparatus. The length of time required for the birds to habituate to the apparatus varied among individuals but occurred over the course of 1–4 habituation sessions across 1–2 days. Crows received habituation trials until they were comfortable approaching the apparatus. After habituation to the platform apparatus, the crows were trained with this apparatus without the 90mm tube on top. Here, crows had to push the platform with their beaks to gain food placed out-of-reach on the platform. Crows initially learnt to take food from the box when the platform was open and then closed. Once, they were confident at taking food from the box, a small bit of meat was placed between the magnets holding the platform and another piece of meat was placed out of reach of the crows. The birds’ pecking at the trapped meat caused the platform to collapse and allowed the crows access to both pieces of meat. As the birds became more comfortable collapsing the platform, the size of the meat placed between the magnets was reduced. When the birds were confidently collapsing the platform, the meat between the magnets was removed and the birds had to continue to push the platform with their beaks to retrieve the inaccessible meat. As in von Bayern *et al*. [12], once crows had pushed the platform with their beak to gain the reward 30 times they began the experiment.

### Block dropping experiment

**Fig 2:**
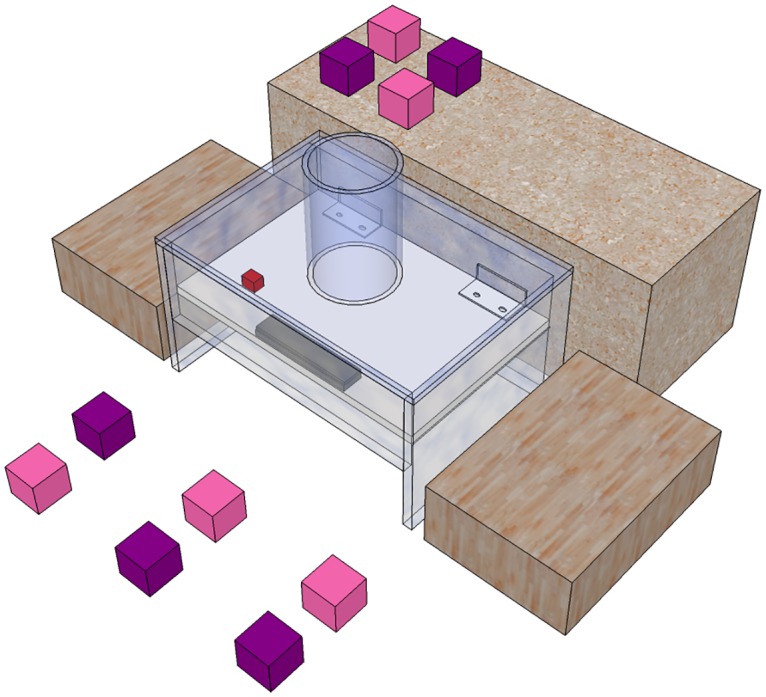
Set up of the blocks and platform apparatus during test trials. Ten blocks (five light and five heavy) were placed in pairs around the platform apparatus.

During testing, the tube was placed back on the platform apparatus and, in each trial, 10 blocks, 5 of each colour were arranged around it, with one block of each colour placed in alternating pairs (Fig 2). Within each pair the position of each block was randomised across trials. While the heavy objects were of sufficient weight to trigger the platform apparatus if dropped down the tube, the light objects were not. A test trial began when a crow landed on the table and ended when the crows got the food or after 3 minutes. Crows were given 3 trials initially. At the end of these 3 trials, if the crows had not solved the task, they were given another 5 trials of pushing the platform with their beak. They were then given an experimental trial. If they did not drop a block again, this pattern was then repeated a further time before testing ended. For crows that did solve the task, testing ended once they had gained the reward by dropping the block in 10 trials.

### Stone dropping training and preference test

Crows that did not complete the experiment were subsequently given training where they learnt to drop a stone down the tube. Training consisted of the crows learning to nudge the stone from the edge of the tube onto the platform. Initially meat was placed on the stone so that, as the crows took the meat, the stone fell into the tube. Over time, as the crows learnt to nudge the stone, the stone was placed progressively further from the tube. When the crows were reliably nudging the stone onto the platform, the stone was placed on the ground so that the crows had to pick it up and drop it in. Once the crows had picked up the stone from the ground and used it to gain the reward 10 times, they were then given the experimental test again, to see if they had developed a preference for the heavy block after learning to stone drop.

## Results

### Object handling pre-test

In the object handling pre-test, birds chose to pull the light blocks out of their tube slightly more often than pulling blocks from the tube containing heavy blocks, doing so in 128 of the 240 trials given (20 trials per bird). This preference was not significant (Binomial test *p* = 0.167; Table 1). Individually, only one bird had a significant preference for either block; picking the heavy block 15 times out of 20 (Binomial test *p* = 0.041.) The other birds had no significant preference for either block (see Table 1 for details.)

### Block dropping experiment

During the block dropping experiment, only two of the twelve crows produced the target block dropping behaviour. On her second trial, D4R dropped one light block down the tube, and then dropped one heavy block, triggering the platform. Across the 10 experimental trials this bird dropped the heavy object 12 times and the light object 22 times and so showed a non-significant trend to drop the light block (Binomial test *p* = 0.062, see S1 Video for example). D3B dropped the light object once on the first trial and then stopped stone dropping, though he did interact with the blocks on a further 5 trials. During these trials he interacted significantly more with the light block than the heavy block (18/24 contacts Binomial test (*p* = 0.012). The remaining ten birds did not drop the blocks down the tube across the experiment, though seven individuals did interact with them. Nineteen of the 30 total contacts with the blocks were with the light one (Binomial test *p* = 0.100). Individual crow interactions are summarized in Table 2.

**Table 2:** Summary of crows’ interactions with blocks during test.

Identity	Adult or Juvenile	Sex	No. of Interactions		No. of blocks dropped into tube	
			Heavy	Light	Heavy	Light
D4B	Adult	Male	0	0	0	0
D4R	Juvenile	Female	13	26*	12	22
D3R	Juvenile	Female	0	1	0	0
D3B	Juvenile	Male	6	18*	0	1
RWY	Adult	Male	2	1	0	0
Sort	Adult	Male	0	0	0	0
Svart	Juvenile	Male	2	0	0	0
Den	Juvenile	Male	3	10*	0	0
Black	Juvenile	Male	3	3	0	0
Nero	Adult	Male	0	3	0	0
Preto	Adult	Male	0	0	0	0
Noir	Adult	Female	1	1	0	0

The number of times the crows interacted with the blocks and the number of blocks dropped into the tubes by the crows

*p<0.05

### Stone dropping training and preference test

In the subsequent test, all of the birds that did not complete the experiment learnt to stone drop after shaping. All of these birds then showed a significant preference for the heavy block after having successfully stone dropped 10 times, with three crows scoring 10/10 immediately (see Table 3 for details). As a group the crows showed a significant preference to drop the heavy block, choosing it on 125/140 trials (Binomial test *p* <0.001).

**Table 3:** Summary of crows’ post-training first choice preferences.

Identity	Adult or Juvenile	Sex	No. of trials	No. of heavy first choices
D4B	Adult	Female	10	9*
D3R	Juvenile	Male	10	9*
D3B	Juvenile	Male	20	18***
RWY	Adult	Male	10	10***
Sort	Adult	Male	10	10***
Svart	Juvenile	Male	20	17**
Den	Juvenile	Male	10	9*
Black	Juvenile	Male	10	9*
Nero	Adult	Male	20	15*
Preto	Adult	Male	10	9*
Noir	Adult	Female	10	10***

The number of trials in which the crows first picked up the heavy block after completing stone dropping training. Birds were initially given 10 trials. If the crows chose the heavy block significantly more than expected by chance (9/10), testing stopped. If they did not, a further 10 trials were given.

*p<0.05

**p<0.01

***p<0.001

## Discussion

The two birds in our study that spontaneously dropped blocks onto the platform to release food did not choose to drop heavy blocks onto a platform and so clearly did not attend to information on object weight (the weight inattention error). One bird dropped the stone only once, likely because the initial dropping of the light block was not followed by reward. The other crow continued stone dropping for the 10 trials of the experiment, without showing any preference for heavy blocks. In contrast, the ten birds that did not complete the experimental task all showed significant preferences for the heavy block after being trained to stone drop. These birds’ behaviour demonstrates that the errors made by the two crows who innovated block dropping were not due to the crows being unable to detect a difference in weight between the two blocks provided, or being unable to inhibit responses towards the lighter blocks. After experience, these crows discriminated between the blocks and were able to immediately inhibit actions towards the non-functional one with three crows scoring 10/10 at this task. Instead, it seems the crows lacked the necessary experience to know that block weight was an important piece of information that needed to be attended to. With experience of stone dropping they did attend to weight, and so made functional choices. Our findings therefore demonstrate that attention to weight is not *necessary* for the production of seemingly ‘insightful’ stone-dropping in New Caledonian crows. Instead, these crows make a specific information processing error when creating spontaneous stone dropping: the weight inattention error.

The proportion of birds who showed spontaneous stone-dropping was lower in our experiment than the original von Bayern *et al* experiment [12]. One possible reason for this difference is that the crows in the original study may have learned to insert their beak into the tube during the stone dropping phase due to having to insert their beak into a 3cm tube in order to collapse the platform in the training phase. In contrast, in our study, the tube was removed from the apparatus when the birds had to collapse the platform with their beak. While this small difference in apparatus design should have had no effect on innovation rates if the crows were using an understanding of force, it could have if stone dropping is innovated through simpler mechanisms, as our results here suggest. Another possible reason for the difference in the number of birds which innovated stone dropping in the two experiments is that two of the three birds that spontaneously stone dropped in the von Bayern *et al* experiment were raised in captivity. In a similar manner to the “captivity bias” seen in apes, where captive apes show greater use of tools than wild apes [32], it is possible that these captive crows experienced reduced task demands compared to the wild birds in our study. In particular, they may have devoted less of their cognitive resources to vigilance behaviour, due to their lack of experience of life in the wild and thus found the task easier to solve.

While stone dropping was only innovated by two of the twelve birds tested, these results do suggest that the anecdote reported in von Bayern *et al* [12], of a crow dropping a feather down the tube, was not due to play or a lack of alternative options, but reflected a limit in their understanding of the task. The presence of the weight inattention error suggests that the beak pushing experience given to the crows in our study did not lead to them developing a full causal understanding of the stone dropping task. Clearly, an understanding of force is not necessary for New Caledonian crows to spontaneously solve this problem. This raises the possibility that these crows lack the ability to reason about invisible forces more generally, though given the suggestive evidence that this species can reason about hidden causal agents ([33]; but see [34–37]), and the weight discriminations made by birds trained to stone drop here, further research is required. Caution is also required because of the role that ontogeny and experience may play in the development of an understanding of force. While we tested 12 birds, of which 6 were adult, only two juveniles innovated stone dropping. While these two juveniles both made the weight inattention error, it is possible that if an adult had innovated stone dropping, it may have attended to weight. Similarly, it is possible that, if the crows had had more experience of force, either through increased experience of beak pushing, or from having to attend to the force of their actions in other contexts, they might not have made the weight inattention error. Thus further work is required before we can make any strong conclusions about the crows’ use of an understanding of force outside of the specific context of innovating stone dropping, where it is clear that such understanding is not necessary for this ‘insightful’ behaviour to emerge.

It is still unclear exactly what New Caledonian crows are thinking when innovating stone dropping. One possibility is that the crows pick up objects close to the apparatus and insert them into the tube in an attempt to increase their reach [38]. When they find the block is not a sufficiently long tool they drop it, thus triggering the platform. Another possibility is that the crows have an abstract concept of contact, in that they learn that contact between their beak and the platform leads to reward, and then generalise this relationship to objects external to their body. A third possibility is that the crows have a causal concept of contact, where they understand that contact between their beak and the platform causes a specific effect: that the platform will collapse and so lead to the release of food. Recent results suggesting that New Caledonian crows are not capable of producing causal interventions provide some evidence against this third possibility [39], though research is clearly needed to test between these possibilities.

Our findings show how the signature-testing approach can allow us to make stronger inferences about cognition than searching for ‘insight’. By designing experiments that explicitly search for key cognitive signatures it is possible to constrain the types of cognitive mechanism that could potentially be producing a behaviour. This can allow us to rule out cognitive mechanisms that do not fit the patterns of observed signatures generated by a non-human animal. It also allows us to make stronger inferences about whether different kinds of minds think in the same way. Searching for the weight inattention error in children and primates, for example, would be a first step in examining how similar the cognition of these groups is to corvids during ‘insightful’ problem solving. Such research offers a highly promising avenue for exploring the degree to which intelligence evolves in a convergent manner across distantly related species.

## Supporting Information

S1 Data Set Data set for the birds’ preferences and interactions with the heavy and light blocks. (XLSX)

S1 Video Example of the weight inattention error. Video clip showing D4R showing the weight inattention error. The subject initially drops a light block into the apparatus (which is too light to collapse the platform) before dropping in a heavy block and causing the platform to collapse.
(3GP)
